# A Validation Study of the Rainbow Model of Integrated Care-Measurement Tool for Patients in China

**DOI:** 10.5334/ijic.5603

**Published:** 2021-04-19

**Authors:** Xin Wang, Stephen Birch, Lijin Chen, Yixiang Huang, Pim Valentijn

**Affiliations:** 1 School of Public Health, Health Development Research Center, Sun Yat-Sen University, Guangzhou 510080, China; 2 Centre for the Business and Economics of Health, University of Queensland, Australia; 3 Department of Health Services Research, Care and Public Health Research Institute (CAPHRI), Faculty of Health, Medicine and Life Sciences, Maastricht University, The Netherlands; 4 Integrated Care Evaluation, Essenburgh, Hierden, The Netherlands

**Keywords:** integrated care, measurement tool, patients with diabetes, primary care

## Abstract

**Introduction:**

The original Rainbow Model of Integrated Care Measurement Tool (RMIC-MT) is based on the Rainbow Model of Integrated Care (RMIC), which provides a comprehensive theoretical framework for integrated care. The aim of this paper is to modify the original patient version of the RMIC-MT for the Chinese primary care context and validate its psychometric properties.

**Methods:**

The translation and adaptation processes were performed in four steps, forward and back-translation, experts review and pre-testing. We conducted a cross-sectional study with 386 patients with diabetes attending one of 20 community health stations in the Nanshan district. We analyzed the distribution of responses to each item to study the psychometric sensitivity. Exploratory factor analysis with principal axis extraction method was used to assess the construct validity. Confirmation factor analysis was used to evaluate model fit of the modified version. Cronbach’s alpha was used to ascertain the internal consistency reliability.

**Results:**

During the translation and adaptation process, all 24 items were retained with some detailed modifications. No item was found to have psychometric sensitivity problems. Five factors (person-centeredness, clinical integration, professional integration, team-based coordination, organizational integration) with 15 items were determined by exploratory factor analysis, accounting for 53.51% of the total variance. Good internal consistency was achieved with each item correlated the highest on an assigned subscale and Cronbach’s alpha score of 0.890. Moderately positive associations (r≥ 0.4, p<0.01) between the score of the scale and these correlations indicate good construct validity.

**Conclusions:**

The results showed initial satisfactory psychometric properties for the validation of the Chinese RMIC-MT patient version. Its application in China will promote the development of people-centered integrated primary care. However, future studies with diverse samples crossing regions would be needed to test its psychometric properties for the various Chinese primary care contexts.

## Introduction

Health systems worldwide are facing demographic and epidemiological transitions marked by population aging and rising prevalence levels of chronic disease and disability [1,2,3]. The World Health Organization (WHO) acknowledges integrated care in its vision and global strategy for health care delivery to address the challenge [4,5]. Many people-centered integrated care programs have been initiated and implemented in high-income countries, which have indicated that integrated health care delivery improves the performance of health systems by improving quality of care, improving patients satisfaction and reducing costs per capita of care [6,7]. In 2016, the report Deepening health reform in China was published jointly by the WHO, the World Bank and the Chinese government, proposing strengthening health care in China through a tiered health-care delivery system in accordance with a people-centered integrated care model [8]. In April 2017, the General Office of the State Council issued a Guideline for constructing Medical Consortia, thus, medical consortia in a county/district became the main strategy for achieving People-Centered Integrated Care in China [9]. Despite the increasing number of established medical consortia in the last three years, care delivery lacks measurement tools specializing in integrated care [10].

International research implied a lack of valid and reliable tools for measuring care integration. A comprehensive systematic review conducted by Bautista et al, which identified 209 instruments to measure integrated care, has shown that most of the instruments have limitations [11]. Firstly, the psychometric properties (e.g. validity and reliability etc.) of most instruments are of low to moderate quality. Secondly, only a few instruments measure integrated care from comprehensive dimensions. Majority of existing instruments contains scales to assess the people-centered care, clinical integration dimensions, only a few instruments contain scales to assess the professional, organizational, and functional integration dimension, and none of the instruments measures normative or system integration [12]. Additionally, key dimensions were measured in either patients or health care providers but very few (8%) were measured in both groups. Research measuring care integration from the perspective of both patient and providers demonstrated a gap of perception between the two groups [12,13]. Providers perception mainly reflect structure and process of care integration. Meanwhile, as the receiver of health care, patients’ experience highly reflects process and outcome of care integration. Suter et al identified 114 instruments, but over half of these instruments were self-reports from questionnaires without psychometric assessments or theoretical framework [13]. As systematic review by Martin S.L. et al also revealed that most methods for measuring integrated healthcare delivery lack information regarding validity and reliability [14]. In order to evaluate integrated care programs, a valid and reliable tool containing comprehensive types of integration under a theoretical framework is required.

Some researches indicated that the diversity of existing low-quality instruments was due to lack of clarity in the concepts and methodologies of integrated care being used [15]. To address the wide range of definitions and absence of a universal framework, the Rainbow Model of Integrated Care (RMIC) was developed, conceptualizing different dimensions of integrated care into a unified model [12]. RMIC was developed based on a literature review and two international Delphi studies [16]. It distinguishes four integrated care dimensions (clinical integration, professional integration, organizational integration, system integration), two enablers (functional integration, and normative integration) at micro- meso- and macro-levels, two guiding principles of integration (person-focused care and population-based care), and three interrelated outcome dimensions (population health, experience of care and cost). More importantly, a measurement tool, RMIC-MT, was developed based on the RMIC, more than 300 integrated care instruments and two international Delphi studies. To measure integrated care comprehensively, the RMIC-MT was divided into a patient version and a provider version by its developer, which shows both providers’ and patients’ perception.

This preliminary version of the RMIC-MT, both patient and provider version, has been tested in the Netherlands, Australia, and Singapore [14,17,18,19]. An international validation of the RMIC-MT across 19 countries has been conducted, which showed that the RMIC-MT is a valuable psychometric tool for evaluating integrated care initiatives in various countries [20]. Pilot validation of the provider version and patient version RMIC-MT were conducted in Chinese primary care systems in 2018, targeting on different populations and with different sampling and data collection methods. The validation of the provider version showed initial satisfactory psychometric properties [21]. This study aims to validate the RMIC-MT patient version in the context of the Chinese integrated primary care system. Application of this tool could promote regular evaluation of integrated care and further implementation of integrated health systems in China.

## Methods

**Figure 1:**
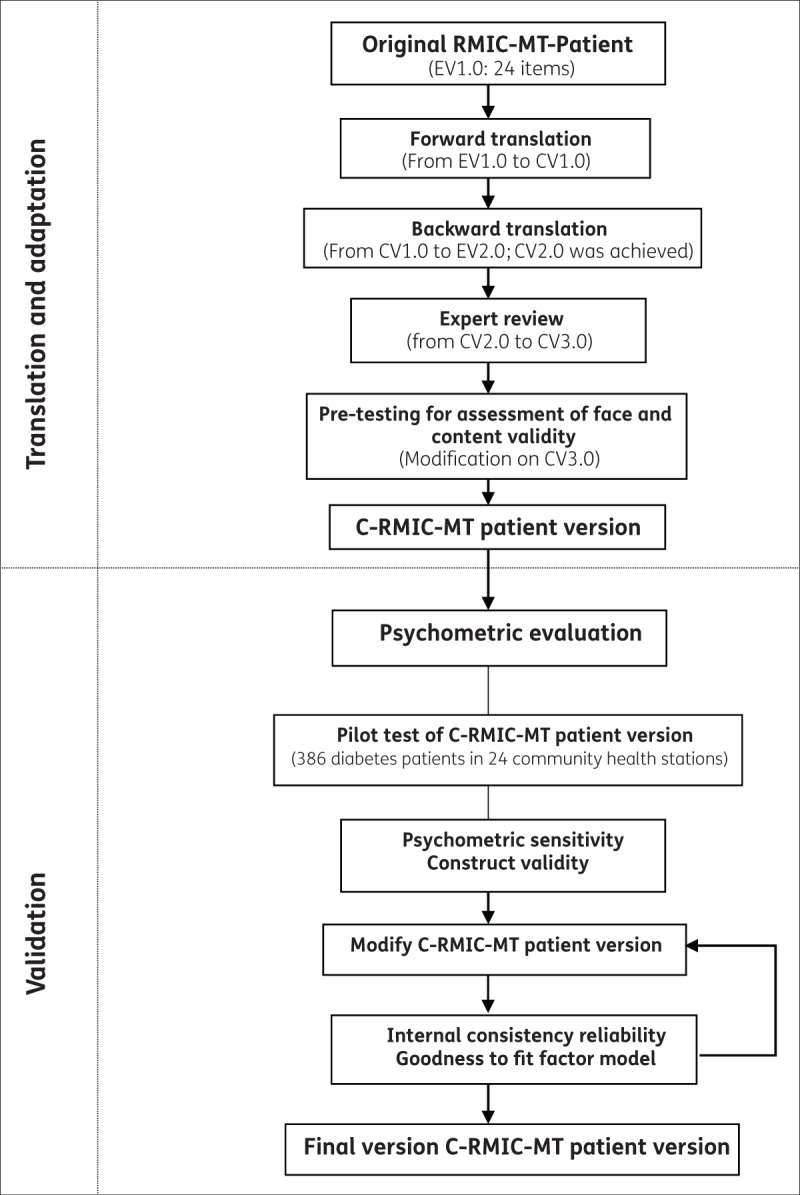
Study design. Note: RMIC-MT is short for Rainbow Model of Integrated Care-Measurement Tool. C-RMIC-MT is short for Chinese version Rainbow Model of Integrated Care-Measurement Tool.

The RMIC-MT patient version, which was tested in the international validation study, was validated in the Chinese primary health systems [22]. The validation was conducted in two phases, instrument translation and adaptation, instrument validation (see ***Figure 1***).

### Study Variable and Scoring of the Instrument

According to dimensions in the Rainbow Model, the RMIC-MT patient version assessed how patients experienced care integration with 24 items in four dimensions: person-centeredness, service integration, professional integration, and organizational integration [23]. All items are answered on a 5-point Likert scale (i.e. never, rarely, sometimes, often, always). The total score of the instrument is computed by summing scores on all 24 items, with a maximum score of 120 points. Patients were also asked to rate coordination, perceived quality of care and their ideal involvement on 10-point scale ranging from very poor (1) to excellent (10). In addition, the sociodemographic characteristics (age, gender, marital status, level of education, health insurance enrollment, work status, income and self-reported health status) and health-related characteristics (history, complication, service utilization) of participants were also collected.

### Instrument Translation and Adaptation

A four-step systematic approach for translation and adaptation of the instrument was adopted: forward translation, backward translation, adaptation, and assessment of content validity [24].

#### Forward and backward translation independently

Two postgraduate students with Chinese as their first language, and majoring in health policy, independently translated the RMIC-MT patient version into Chinese. The lead author reviewed the two forward-translations and discussed with the translators until agreement achieved on a reconciled Chinese version 1.0 (CV1.0). The CV1.0 was translated back into English by two PhD candidates with English as first language and majoring in health policy, who had never read the original English version (EV1.0). The backward-translations and EV 1.0 were compared and discussed by the lead author (XW) and the two translators with the aim of reaching satisfactory equivalence between EV2.0 and EV1.0. CV1.0 was modified to CV2.0 after the discussion.

#### Adaptation by expert review

Semi-structured interviews were conducted independently with four university based Chinese experts working on primary care to obtain their reflections on the suitability of the CV2.0 for use in Chinese primary health systems research. The final CV3.0 was obtained based on changes arising from the experts’ reflections.

#### Assessment of content validity by pre-testing

Pre-testing was conducted with 18 patients with diabetes. Through a face-to-face interview, each patient was asked to review each item on the CV3.0, and comment on wording, relevance and user burden of the items. The relevant items were rated as 1 and the un-relevant items rated as 0, and a content validation index was calculated to test content validity of the CV3.0. After the pre-testing phase, two researchers (XW and PV) discussed and agreed on the final C-RMIC-MT patient version.

### Instrument Validation

#### Design

A pilot study was conducted for psychometric assessment of the C-RMIC-MT patient version in Nanshan district, Shenzhen city. Economy of Nanshan district ranked first among all 2846 counties/districts of China in 2020. Nanshan district established a medical consortium with the purpose of promoting health care integration in 2017. In the medical consortium, there are five hospitals and 79 community health stations (CHSs). Competence of care provision in CHSs was enhanced by strengthening Cooperation between hospitals and CHSs in the medical consortium strengthened continuity and integration of care, especially for patients with chronic diseases. In regard to patient sampling, we chose patients with type 2 diabetes. Patients with chronic conditions are in need of health services from multiple providers and units over time, and hence request the coordinated delivery of care. Diabetes, as one of the two chronic conditions, has been incorporated in the Equalization of Essential Public Health Service program since 2009 to promote integrated care delivery. Patients with diagnosed diabetes who are under case management (health education, one physical examination, and four follow-ups per year) in CHSs were invited to participate in the study as they had experienced care coordination inside and outside CHSs [25].

#### Study Population and Data Collection

The estimated minimal sample was based on the requirement of 10 subjects per item with each C-RMIC-MT questionnaire [26]. Given that the instrument had 24 items, the required sample size was 240. The study included a representative sample bigger than the size recommended by the statistical analysis. Half CHSs (40/79) under the unified management of Nanshan medical consortium were selected randomly. Ten patients were randomly selected from list of all patients under diabetes management in each CHS, and invited to participate in the study. Patients were considered eligible to participate in the study if they met the following criteria: 1) aged 18 years old; 2) treated with diagnosed type 2 diabetes for 90 days or longer; 3) able and willing to provide informed consent; 4) able to complete a face-to-face questionnaire with trained investigators. All participants gave written informed consent before recruited into the study. Data were collected in July 2018 with the support of the Nanshan medical consortium.

#### Data Analysis

Questionnaires with more than 30% missing data were excluded from the analysis. Data were entered and cleaned before the analysis. All statistical analyses were done by SPSS 23.0 and Amos 24.0. A P-value <0.05 was considered statistically significant.

##### Psychometric sensitivity

The distribution of responses to each item was analyzed for the study of psychometric sensitivity. Items with skewness value >3 and kurtosis value >7, or items with floor or ceiling effects of >75% of respondents, were considered for deleting as a result of psychometric sensitivity [27,28].

##### Factor analysis

With a Kaiser-Meyer-Olkin (KMO) value over 0.80 and a significant Bartlett’s test, exploratory factor analysis using the principal axis factoring extraction method was conducted to assess the underlying structure of the C-RMIC-MT patient version [29,30]. Exploratory factor analysis in this study followed the description by Brown [31,32]. The number of factors was determined by consideration of the eigenvalue (>1), scree plot, and interpretability of the factor. More importantly, the factors retained were guided theoretically. Names were used for each identified factor based on the dimensions of the RMIC. Items that cross-loaded on more than one factor were grouped into the factor that was most closely related conceptually. Items with poor factor loading (<0.60) were removed from the questionnaire [33]. In addition, a structural equation model with maximum likelihood was used to evaluate the explorative factor analysis model fit by using the standard fit indices: root-mean-square error of approximation RMSEA (≤0.06), standardized root-mean-square residual (SRMR) (≤0.08), comparative fit index (CFI) (≥0.80), Tucker-Lewis index (TLI) (≥0.80).

##### Internal consistency reliability

Based on potential modification in the above two phases, internal consistency reliability was assessed by items-total correlations and Cronbach’s alpha. Items-total correlation coefficients between items within a scale should be r≥0.40 [34]. If Cronbach’s alpha ranged between 0.70 and 0.95, the scale was considered reliable for use in the sample population [35]. Moreover, Pearson’s correlation coefficients were calculated to assess whether each item was correlated the highest on an assigned subscale by correlation of items with the subscale means. Any items that correlated more highly on subscales other than the one to which the items were assigned was eliminated [36].

##### Construct validity

Pearson’s correlations between the scale scores and the overall perceived coordination questions were calculated to assess construct validity. Moderately positive associations (r≥0.40) between the score of the scale and these correlations would indicate good construct validity [37]. Additionally, the two hypotheses were tested based on previous research [38]: 1) patients who have a better coordinated care experience are more satisfied with quality and treatment involvement; 2) each subscale aimed at measuring coordinated care experience is positively and significantly correlated with other subscales.

## Results

### Instrument Translation and Adaption

Satisfactory equivalence between EV2.0 and EV1.0 was achieved after forward- and backward-translation. There were several modifications during the adaptation process as suggested by the experts and patients. In item 11 “My care team knows very well what I think is important when it comes to my care”, “e.g. save cost, reduce pain or don’t bother family members” were added as an explanation for “what”. In item 17 “My care team knows the results of my visits to other doctors.”, “e.g. diagnosis, treatment” were inserted as an explanation for “results”. In item 18 “My care team always asks how my visits with other care providers are going”, “e.g. visit processes and results” were added as explanation for “how…are going”. Additionally, two modifications were made based on the background of the Chinese health system. GPs and public health physicians were included as members of a multidisciplinary team, rather than psychologists and dietitians in item 22 of the original version; replacing “quickly enough” with “within three days” in original item 21 “I can get appointment with specialist in hospitals quickly enough through my care team.” After modification, the scale content validity for the entire instrument was 0.86 in the pre-testing.

### Instrument Validation

Among a random sampled 400 diabetes patients in 40 CHSs, 386 patients participated in the study with a response rate 96.50%. Average age of the participants was 56.13 years old. ***Table 1*** summarizes the sociodemographic and health-related characteristics of the participants.

**Table 1:** Characteristics of participants.


SOCIODEMOGRAPHIC AND HEALTH-RELATED CHARACTERISTICS	N (%)

**Gender**	

Male	248 (64.25)

Female	138 (35.75)

**Marital status**	

Married	356 (92.23)

Others	30 (7.77)

**Level of education**	

Junior technical college	239 (61.92)

Senior technical college	127 (32.90)

Undergraduate and graduate-university	20 (5.18)

**Health insurance schemes**	

None	338 (21.19)

Urban employee basic medical insurance	159 (41.19)

Migrant worker basic medical insurance	44 (11.40)

Urban resident basic medical insurance	60 (15.54)

Health insurance of other provinces	54 (13.99)

**Employment**	

Retired from paid work	93 (24.09)

employed	185 (47.93)

Other options	108 (27.98)

**Income (¥/year)**	

<20,000	157 (40.67)

20,000–50,000	141 (36.53)

50,000–100,000	60 (15.54)

≥100,000	28 (7.25)

**How many years have you had diabetes?**	

<5 years	212 (54.92)

5–10 years	107 (27.72)

>10 years	67 (17.36)

**Do you have diabetes complication?**	

Yes	42 (10.88)

No	344 (89.12)

**Have you signed contract with general practitioner in community health stations?**	

Yes	102 (26.42)

No	284 (73.58)

**How would you describe your health today?**	

Very good and good	292 (75.65)

fair	85 (22.02)

Poor and very poor	9 (2.33)

**How many times did you visit care providers during the last four months?**	

<10 visits	283 (73.32)

10–19 visits	85 (22.02)

≥20 visits	18 (4.66)


#### Psychometric sensitivity

Distribution analysis of responses to each item showed that there was no item with a skewness value >3 or kurtosis > 7 and there were no items had a floor or ceiling effect of >75%, which indicated the adequate psychometric sensitivity of the items.

#### Factor analysis

The KMO value of 0.90 and significant Bartlett’s test met the requirements for factor analysis. In the EFA, four factors yielded eigenvalues >1 accounting for 50.45% of the total variance (***Table 2***). One factor had an eigenvalue <1, accounting for 3.06% of the variance and was included because it was interpretable based on the RMIC. A five-factor solution was obtained. Factor 1 was named ‘clinical integration’ (5 items, 15.29% variance), factor 2 ‘professional integration’ (3 items, 11.02% variance), factor 3 ‘team-based coordination’ (3 items, 9.98% variance), factor 4 ‘organizational integration’ (2 items, 9.44% variance), factor 5 ‘person-centeredness’ (2 items, 7.78% variance). Items 1, 11, 15, 19, 20, 21, 22, 23, 24 were omitted because they had poor factor loadings (<0.60) (***Table 3***). Regarding model fit (15 items, five factors), the following test of significance and goodness-of-fit measures were obtained: RMSEA 0.046, SRMR 0.057, CFI 0.896, TLI 0.885. The model passed majority of goodness-to-fit tests by confirmation factor analysis.

**Table 2:** Eigenvalue and variance contribution rate of each factor.


FACTOR	EXTRACTION SUMS OF SQUARED LOADINGS				ROTATION SUMS OF SQUARED LOADINGS		
	
	TOTAL	% OF VARIANCE	CUMULATIVE %		TOTAL	% OF VARIANCE	CUMULATIVE %

1	7.68	32.00	32.00		3.67	15.29	15.29

2	2.02	8.40	40.40		2.65	11.02	26.31

3	1.42	5.86	46.26		2.40	9.98	36.29

4	1.01	4.19	50.45		2.27	9.44	45.73

5	0.73	3.06	53.51		1.87	7.78	53.51


Extraction method: Principle Axis Factoring.

**Table 3:** Factor analysis C-RMIC-MT patient version (n = 386).


ITEM NO.	CONTENT	ROTATED FACTOR LOADINGS*				

		1. CLINICAL INTEGRATION	2. PROFESSIONAL INTEGRATION	3. TEAM-BASED COORDINATION	4. ORGANIZATIONAL INTEGRATION	5. PERSON-CENTEREDNESS

**1**	Explaining	.41				

**2**	Listening	.69				

**3**	Preference integration	.84				

**4**	Communication	.7224				

**5**	Questioning	.62				

**6**	Shared decision-making	.72				

**15**	Interdisciplinary fragmentation		.54			

**16**	Interdisciplinary contact		.74			

**17**	Interdisciplinary information continuity		.87			

**18**	Interdisciplinary treatment continuity		.86			

**7**	Care continuity in the team			.70		

**8**	Treatment longitudinally in the team			.82		

**14**	Interdisciplinary coordination			.63		

**19**	Accessibility of team care			.33		

**23**	Time management			.06		

**12**	Interdisciplinary communication				.61	

**13**	Interdisciplinary collaboration				.66	

**20**	Appointments					

**21**	Results				.46	

**22**	Multidisciplinary team					

**24**	Accessibility				.46	

**9**	Family circumstances					.80

**10**	Social circumstances					.69

**11**	Needs assessment					.52


* Factor loadings above 0.30 are reported.

#### Internal consistency

The results of the internal consistency analysis indicated that reliability assumptions were adequately met. Item-total correlations exceeded 0.40 for all items. Cronbach’s alpha for the five components, showed good internal consistency among the items (0.75 for the person-centeredness, 0.87 for clinical integration, 0.91 for professional integration, 0.89 for team-based coordination, 0.83 for organizational integration). Correlation matrices of Pearson’s correlation coefficients revealed that each item was correlated the highest on an assigned subscale. The RMIC-MT patient version with 15 items is a reliable scale based on Cronbach’s alpha of 0.89.

#### Construct validity

All subscales of the instrument aimed to measure coordination of care experience and were significantly correlated with other subscales (see ***Table 4***). Patients who experienced better overall care coordination were more satisfied with the quality of care. However, the hypotheses that patients who experience better care coordination show higher ideal treatment involvement was not supported. The ideal treatment involvement score is 5.77 in this study.

**Table 4:** Correlation between subscale scores C-RMIC-MT patient version (n = 386).


VARIABLES	1	2	3	4	5	6	7	8

1. Person-centeredness (item 1–2)								

2. Clinical integration (item 3–7)	.46**							

3. Professional integration (item 8–10)	.26**	.29**						

4. Team-based coordination (item 11–13)	.36**	.57**	.30**					

5. Organizational integration (item 14–15)	.17**	.40**	.23**	.38**				

6. Overall care coordination	.20**	.23**	.30**	.18**	.35**			

7. Quality of care	.22**	.31**	.31**	.19**	.31**	.74**		

8. Treatment involvement	.04	.37	.13**	.15**	.12*	.01	.06	


**. Correlation is significant at the 0.01 level (2-tailed). *. Correlation is significant at the 0.05 level (2-tailed).

## Discussion

### Principal Findings

This study provides the first assessment of the validity and reliability of the C-RMIC-MT patient version. The C-RMIC-MT showed excellent content validity in expert review. The clarity and feasibility of the C-RMIC-MT patient version was assessed by pre-testing with 18 patients. The psychometric properties of the C-RMIC-MT patient version were tested in the pilot study of 386 patients with diabetes. Statistical analyses showed that the reliability and construct validity for the C-RMIC-MT patient version (15 items, 5 subscales) were good. This suggested that the C-RMIC-MT patient version is a valuable tool for evaluating integrated care in Chinese primary care settings.

### Comparison with Other Studies

The factor analysis of the C-RMIC-MT patient version indicated that respondents differentiate between hypothesized dimensions of integrated care (i.e. clinical integration, professional integration, organizational integration, person-centeredness). Differently, care providers participated in validation of the provider version C-RMIC-MT didn’t recognize differences between clinical and organizational integration [21]. The person-centeredness dimension did not meet the eigenvalue criteria of >1. It might be due to that people do not completely understand what person-centeredness means or what they are supposed to do to be responsible for their health, which is also shown by a low ideal involvement score (5.77/10) of decision making in this study [39]. Original items 19–24, which were hypothesized to belong to the organizational integration dimension, were omitted based on low factor loadings (<0.6). Historical relatively weak collaborations among organizations in Chinese primary care system may result in patients’ low recognition and experience of organizational integration [40].

The factor analysis of the RMIC-MT patient version with 17,512 chronic kidney disease in 19 countries (e.g. Argentina, Australia, Chile, France, Germany, Hungary, Italy, Kazakhstan, Lithuania, New Zealand, Poland, Portugal, Romania, Russia, Saudi Arabia, Spain, Sweden, UK and Uruguay) concluded the same domains (person-centered care, clinical coordination, professional coordination, and organizational coordination) of integrated care except for team-based coordination. Most of the variance was explained by clinical integration, which is consistent with results of international study. Bautista et al found clinical integration as the one of the most common dimensions by a systematic review of instruments measuring integrated care [11]. In addition, one more factor, “team-based care”, was determined by the factor analysis which is reasonable in Chinese CHSs. As patients could go to see a doctor in CHSs without appointment, it is unlikely that they will see the same member of their family doctor team all the time. Therefore, coordination in the family doctor team may have effect on patients experience of care integration.

### Strengths and Limitation

This study has three highlights. First, the RMIC-MT focuses on comprehensive dimensions compared with previous instruments. Its adaptation, combined with adaptation of the C-RMIC-MT provider version, could fill the gap in existing instruments in terms of system or normative integration assessments [11]. Second, the instrument was based on thorough translation, adaptation and validation processes. Forward and backward translation, cultural adaptation, pre-test and pilot study were conducted completely, and content validity, reliability and construct validity were assessed strictly. Third, we got the first Chinese instrument to measure integrated care from the perspective of patients with good psychometrics properties in Chinese primary care settings.

However, there were several limitations. First, while the number of respondents met the requirement (over 10 times the item number), only patients with diabetes from the public CHSs in a single district were presented. High homogeneity of the sampled CHSs might influence the validity assessment. Future studies with diverse samples crossing regions or patient groups would be needed to further test the psychometric properties for the Chinese primary care context. Second, while validity of the C-RMIC-MT patient version was addressed in the current study, more research is needed to assess the test-retest reliability and construct validity.

### Implications for Practice

Evidence has shown that integrated care evaluation and feedback would provide an incentive for program improvement [41]. As the first value instrument to assess integrated care in Chinese primary care settings, the C-RMIC-MT patient version can be used as an indicator to monitor the effectiveness of medical consortia and other people-centered integrated care programs. It can reveal whether the levels of integration across dimensions change over time in a longitudinal study. Additionally, it can promote comparisons of patients experience on integrated care across primary health care systems in China and globally. The process and results of its validation implies that it is necessary to strengthen person-centered care and promote patient involvement in clinical decision making.

## Conclusion

In conclusion, we validated the C-RMIC-MT patient version in the context of Chinese primary care systems. The results show that the instrument with 15 items grouped into five dimensions (person-centeredness, clinical integration, professional integration, team-based coordination, and organizational integration) has good validity and reliability. The instrument can be used to measure integrated care in Chinese primary care settings from the perspective of patients, and contributes towards international comparison of integrated care.

## Disclaimer

We certify that the materials reported in this paper is not under consideration for publication elsewhere and its publication is approved by all authors and responsible authorities where the work was carried out.
